# Supplementary material for: Complexome profiling of the Chlamydomonas psb28 mutant reveals TEF5 as an early PSII assembly factor
Source: Plant Cell. 2025 Mar 17;37(6):koaf055. doi: 10.1093/plcell/koaf055 (PMC12164590; doi:10.1093/plcell/koaf055)
Supplement: koaf055_Supplementary_Data [file koaf055_supplementary_data.zip › psb28_interactive.zip!/plots/intensity/qualityPlots/peptides/_Cre17.g745097.t1.1.html]

### Cre17.g745097.t1.1

Trivial name:   
SeqCoverage [median]: 5.20  
Mass: 95.46 kDa  
  
 

| Sequence | MissedCleavage | Mass [Da] | wtIntensity | muIntensity | Score |
| --- | --- | --- | --- | --- | --- |
| LATVLGK | 0 | 700.45 | 0.00 | 1796.91 | 88.73 |
| SGQAPIVK | 0 | 798.46 | 391.17 | 0.00 | 93.37 |
| SGSAITITAAR | 0 | 1046.57 | 0.00 | 4216.15 | 123.86 |
| SLGQETLLR | 0 | 1015.57 | 0.00 | 550.23 | 63.48 |
| TFLAPTDTAVAR | 0 | 1261.67 | 794.35 | 9006.03 | 86.62 |
|  |  |  | 1185.5 | 15569.3 |  |
